# Paciente con insuficiencia suprarrenal por mutación *de novo* en el gen *NR0B1*

**DOI:** 10.1515/almed-2022-0099

**Published:** 2023-03-24

**Authors:** Daniel Bravo Nieto, Alba S. García Fernández, Noelia Diaz Troyano, Marina Giralt Arnaiz, Andrea Arias García, Paula Fernández Álvarez, Ariadna Campos Martorell, Roser Ferrer Costa, María Clemente León

**Affiliations:** Servicio de Bioquímica, Laboratorios Clínicos, Hospital Universitari Vall d’Hebron, Barcelona, España; Unidad de endocrinología pediátrica, Hospital Universitari Vall d’Hebron, Barcelona, España

**Keywords:** hipoplasia adrenal congénita, insuficiencia suprarrenal, NR0B1

## Abstract

**Objetivos:**

La hipoplasia suprarrenal congénita ligada al cromosoma X es una enfermedad rara con base genética conocida, que se presenta con insuficiencia suprarrenal e hipogonadismo hipogonadotrófico y expresión clínica variable.

**Caso clínico:**

Paciente varón, de 26 días, que ingresó con síntomas compatibles con insuficiencia suprarrenal, hiponatremia e hiperpotasemia, requiriendo sueroterapia con suplementos de NaCl y fludrocortisona, consiguiéndose estabilidad clínica. Se descartó la hiperplasia suprarrenal congénita tras la medición de 17-OH-progesterona. El resto de hormonas estaban dentro de los intervalos de referencia, salvo la hormona adrenocorticotrópica (ACTH), sensiblemente por encima, y la aldosterona, por debajo. En los siguientes análisis se estudiaron los ácidos grasos de cadena muy larga para descartar adrenoleucodistrofia, el gen CYP11B2 (aldosterona sintasa), y se realizó una RMN para descartar otras alteraciones morfológicas. Todas estas pruebas resultaron normales. Finalmente, tras detectar déficit de cortisol en una analítica, se realizó un estudio genético más amplio donde se describió una mutación en el gen NR0B1, estableciéndose el diagnóstico de hipoplasia suprarrenal congénita.

**Conclusiones:**

La hipoplasia suprarrenal congénita es una enfermedad de diagnóstico complejo debido a la variabilidad en la expresión clínica y el grado de alteración de las pruebas de laboratorio, requiriéndose un seguimiento exhaustivo y la realización de pruebas genéticas para llegar al diagnóstico.

## Introducción

La hipoplasia suprarrenal congénita (HAC) ligada al cromosoma X (OMIM 300200) es una enfermedad rara que suele presentarse con insuficiencia suprarrenal (ISR) en la infancia temprana e hipogonadismo hipogonadotrópico (HH), normalmente detectado en la pubertad. La expresión clínica es variable, detectándose distintos niveles de afección en el metabolismo de glucocorticoides, mineralocorticoides y hormonas sexuales.

Se ha demostrado la implicación del gen *NR0B1* (Xp21.2), que codifica la proteína DAX1, implicada en transducción de señales y expresada en corteza adrenal, gónadas, hipotálamo e hipófisis. Se han descrito más de 100 variantes con distintos efectos fenotípicos, siendo los más habituales la ISR, HH y alteraciones de la espermatogénesis ​ [1, 2]​. El diagnóstico genético precoz de la HAC puede disminuir la morbimortalidad, garantizar el reemplazo hormonal y permitir el asesoramiento genético en los familiares.

Presentamos el caso de un paciente que ingresó con síntomas de hipoaldosteronismo aislado, y durante el seguimiento se detectaron otras alteraciones que llevaron al diagnóstico de HAC ligada a X.

## Caso clínico

Paciente varón de 26 días, derivado de otro centro por síndrome poliúrico con deshidratación, pérdida del 9% del peso al nacer, inapetencia y somnolencia.

No presentaba antecedentes perinatales de interés: segunda gestación, nacido por cesárea a las 39 semanas y 4 días, peso: 4020 g (p95), talla: 50,5 cm (p56) y cribado neonatal normal.

En la analítica del hospital de origen destacó: Na^+^ 126 mmol/L (valores de referencia: 136–146 mmol/L), y K^+^ 6,89 mmol/L (3,90–6,00 mmol/L). Se inició sueroterapia con suero glucosado al 5% y suplementos de NaCl, con mejora clínica. Al pasar a suplementos orales, volvió a agravarse la hiponatremia, y a las 72 h se reinició el tratamiento endovenoso y se añadió al tratamiento fludrocortisona. Se solicitó estudio hormonal, que mostró valores elevados de ACTH de 966 pg/mL (5–49 pg/mL), concentraciones de aldosterona disminuidas, y 17-OH progesterona no sugestiva de HSC.

Fue entonces derivado a nuestro centro. Se amplió el estudio hormonal, encontrándose alteraciones similares, junto a niveles de cortisol dentro de los valores de referencia. Tanto la ACTH como el cortisol muestran ritmos circadianos; las muestras para estos análisis se tomaron siempre a primera hora de la mañana.

La evolución analítica del paciente se presenta en la Tabla 1.

**Tabla 1:**
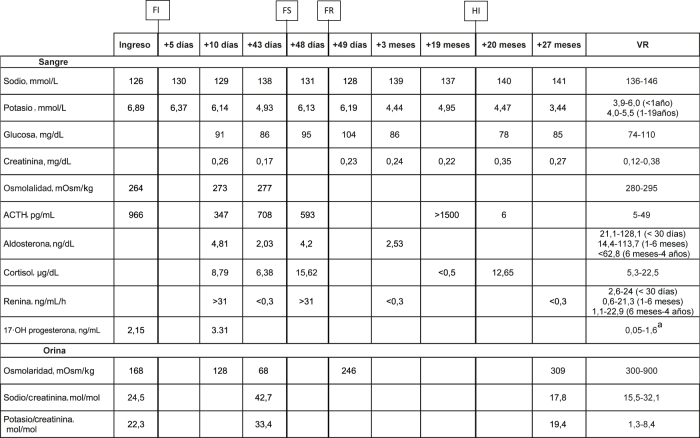
Evolución analítica del paciente.

^a^Una elevación de 17-OH progesterona por encima de 10 ng/mL se considera indicativa de hiperplasia suprarrenal congénita por deficiencia de 21-OH hidroxilasa [10]. FI, inicio fludrocortisona; FS, suspensión fludrocortisona; FR, reinicio fludrocortisona; HI, inicio hidrocortisona; VR, valores de referencia.

En nuestro hospital evolucionó favorablemente con aportes endovenosos de NaCl (hasta 2 mEq/kg a 1,2 cc/12 h), que posteriormente se administraron por vía oral (3 mEq/kg), y con fludrocortisona. El paciente se mantuvo con poliuria, entre 4–6 cc/kg/hora. Se objetivó una recuperación ponderal de 350 g en 6 días. Fue dado de alta con controles periódicos.

A las 6 semanas de la primera visita, se constató una normalización de los valores de sodio y potasio. Se suspendió entonces el tratamiento con fludrocortisona para valorar las hormonas sin interferencias por medicación, previa instrucción a los padres de acudir a urgencias si detectaban empeoramiento clínico. En ese momento los valores de ACTH continuaban siendo elevados (708 pg/mL), pero el resultado se obtuvo días después de haber decidido suspender el tratamiento.

Cinco días después de la suspensión de fludrocortisona, los padres afirmaban notarlo más inactivo e inapetente. La analítica mostraba de nuevo hiponatremia (Na+ 131 mmol/L), y había perdido 300 g de peso. Respecto al estudio hormonal, se constató de nuevo una marcada elevación de ACTH (593 pg/mL), con cortisol inadecuadamente normal para el estado de hiponatremia, aldosterona disminuida, y actividad de renina plasmática elevada (>31 ng/mL/h) (0,6–21,3 ng/mL/h).

Ante la sospecha de ISR primaria, y descartada la HSC por los valores normales de 17-OH-progesterona, se estudiaron otras posibles causas congénitas. La medición de ácidos grasos de cadena muy larga, para descartar adrenoleucodistrofia, dio resultado normal. La RMN mostró glándulas suprarrenales sin alteraciones de tamaño y morfología. Ante la sospecha de una forma de ISR con déficit aislado de mineralocorticoides, se solicitó estudio del gen *CYP11B2*, (previa firma del consentimiento informado) que codifica la aldosterona sintasa y ha sido relacionado con alteraciones en la acción de la aldosterona ​ [3, 4]​. No se identificó ninguna variante patogénica ni de significado incierto en toda la región codificante de *CYP11B2* ni en las regiones intrónicas adyacentes. El paciente se mantuvo clínicamente estable los meses siguientes con el tratamiento con fludrocortisona.

En la analítica de control de los 19 meses, y por aumento de pigmentación cutánea, se detectó déficit de cortisol (<0,5 μg/dL) (5,3–22,5 μg/dL). La sospecha diagnóstica pasó a ser ISR completa, y se solicitó el estudio de genes asociados con déficit familiar de glucocorticoides. El panel NGS ha sido desarrollado en el laboratorio de genética de los laboratorios clínicos del Hospital Universitari Vall d’Hebron en base a las alteraciones más prevalentes que afectan a la función suprarrenal. Incluye los genes *CYP11A1, STAR, NR0B1, MC2R, MRAP, CYP17A1, MCM4, NNT, TXNRD2.* Se secuenciaron utilizando el secuenciador MiSeq (Illumina, Inc.) En *NR0B1* se encontró en hemicigosis un cambio de citosina por adenina en la posición 323 del exón 1 (c.323C>A p. (Ser108*)). Esta variante predice un cambio de arginina por un codón de stop en el aminoácido 108 de la región “4 X 67 AA tandem repeats” y la aparición de una proteína truncada o ausencia de proteína. Las variantes de pérdida de función en *NR0B1 s*on un mecanismo de patogenicidad establecido en individuos con HAC ligada a X.​ [1, 2]​.

La variante encontrada no se había identificado hasta ahora en las bases de datos consultadas: SNP, 1000genomes, ExomeVariant Server, Exome Aggregation Consortium, LOVD y HGMD. ​ [5], [6], [7], [8], [9]​.

Según los criterios para la interpretación y clasificación de variantes de American College of Medical Genetics y Genomics Association for Molecuar Pathology, la variante c.323C>A p. (Ser108*) se considera patogénica.

Se realizó un estudio genético a los padres y ninguno era portador, por lo que se presupone un origen *de novo* en el paciente.

Tras detectar el déficit de glucocorticoides, se añadió hidrocortisona al tratamiento, consiguiendo estabilidad clínica y analítica durante las siguientes revisiones, con buen desarrollo pondoestatural.

## Discusión

La ISR tiene diversos orígenes y manifestaciones clínicas. Puede originarse a nivel suprarrenal (ISR primaria), hipofisario (secundaria) o hipotalámico (terciaria). Además, pueden existir formas parciales, con déficit aislados de glucocorticoides, mineralocorticoides, o andrógenos. Esta variabilidad hace compleja la interpretación de los análisis bioquímicos y hormonales.

La HAC puede presentar una clínica variada, apareciendo de forma simultánea o consecutiva las alteraciones gluco y mineralocorticoide, y con distintos grados de afección. Sus síntomas iniciales pueden ser indistinguibles de la HSC [10, 11]. El caso descrito se presentó inicialmente con síntomas de hiponatremia en un contexto sin antecedentes de interés. El cribado neonatal fue normal, pero en la Comunidad de Cataluña no se incluye el cribado de la HSC. La afección más prevalente en pacientes que presentan, normalmente en los primeros meses de vida, clínica de pérdida de peso, poca apetencia y deshidratación con pérdida de sodio e hiperpotasemia, es la HSC por deficiencia de 21-hidroxilasa. En el caso descrito, las concentraciones normales de 17-OH progesterona para la edad postnatal no eran concordantes con esta hipótesis [12].

Dado que la elevación de la ACTH no llegaba a las concentraciones asociadas al déficit de glucocorticoides (habitualmente >1000 pg/mL), [13] que las concentraciones de cortisol estaban dentro del intervalo de referencia, el desarrollo de los genitales externos era normal, y sólo la aldosterona estaba disminuida, la sospecha inicial fue deficiencia aislada de mineralocorticoides.

La evolución del caso mostró como los déficits hormonales pueden manifestarse de forma gradual: la función mineralocorticoide fue la primera en estar alterada, mostrando síntomas desde las 3 semanas de vida, mientras que la alteración de la función glucocorticoide no se detectó hasta los 19 meses. La elevación de ACTH en estos casos puede ser un precedente a la detección de déficit de glucocorticoides, sugiriendo la existencia de HAC. A nivel clínico, la pigmentación de las mucosas puede afianzar la sospecha, al ser indicativas de valores crónicamente elevados de ACTH, ya que ésta deriva de la proopiomelanocortina, una prohormona que se escinde en ACTH y melanotropina.​ [1]​.

La elevación de la actividad de la renina plasmática en el caso descrito se interpreta como una respuesta compensatoria al déficit de aldosterona, ya que el estímulo de baja tensión arterial no llega a ser corregido. Esta elevación desaparece (incluso el valor llega a ser indetectable) tras el tratamiento con fludrocortisona. La secreción de aldosterona está regulada principalmente por el sistema renina-angiotensina-aldosterona, pero también está modulada, de forma menos potente, por la ACTH.

## Puntos claves

– Ante una sospecha de alteración de la función suprarrenal, si los síntomas y las pruebas iniciales no son concluyentes, el seguimiento analítico y clínico es esencial para poder llegar al diagnóstico.
– En estos casos, los valores elevados de ACTH y renina plasmática, junto a valores normales o bajos de cortisol y aldosterona, podrían ser indicativos de ISR. Las concentraciones de andrógenos y de 17-OH progesterona ayudarían a orientar el diagnóstico hacia hipo-o hiperplasia suprarrenal congénita.
– Como en otros casos descritos, la presentación gradual de los síntomas dificulta el diagnóstico y es necesario el estudio genético para establecer un diagnóstico definitivo, así como identificar a las mujeres portadoras y ofrecer consejo genético.
